# Retinoid X receptor and peroxisome proliferator-activated receptor-gamma agonists cooperate to inhibit matrix metalloproteinase gene expression

**DOI:** 10.1186/ar2564

**Published:** 2008-12-01

**Authors:** Peter S Burrage, Adam C Schmucker, Yanqing Ren, Michael B Sporn, Constance E Brinckerhoff

**Affiliations:** 1 Department of Biochemistry, Dartmouth Medical School, North College Street, 7200 Vail Building, Hanover, NH 03755, USA; 2 Department of Pharmacology, Dartmouth Medical School, North College Street, 7650 Remsen Hall, Hanover, NH 03755, USA; 3 Department of Medicine, Dartmouth Medical School, 1 Medical Center Drive, Lebanon NH 03756, USA

## Abstract

**Introduction:**

We recently described the ability of retinoid X receptor (RXR) ligand LG100268 (LG268) to inhibit interleukin-1-beta (IL-1-β)-driven matrix metalloproteinase-1 (*MMP-1*) and *MMP-13 *gene expression in SW-1353 chondrosarcoma cells. Other investigators have demonstrated similar effects in chondrocytes treated with rosiglitazone, a ligand for peroxisome proliferator-activated receptor-gamma (PPARγ), for which RXR is an obligate dimerization partner. The goals of this study were to evaluate the inhibition of IL-1-β-induced expression of *MMP-1 *and *MMP-13 *by combinatorial treatment with RXR and PPARγ ligands and to investigate the molecular mechanisms of this inhibition.

**Methods:**

We used real-time reverse transcription-polymerase chain reaction to measure LG268- and rosiglitazone-mediated inhibition of MMP gene transcription in IL-1-β-treated SW-1353 chondrosarcoma cells. An *in vitro *collagen destruction assay was a functional readout of MMP collagenolytic activity. Luciferase reporter assays tested the function of a putative regulatory element in the promoters of *MMP-1 *and *MMP-13*, and chromatin immunoprecipitation (ChIP) assays detected PPARγ and changes in histone acetylation at this site. Post-translational modification of RXR and PPARγ by small ubiquitin-like modifier (SUMO) was assayed with immunoprecipitation and Western blot.

**Results:**

Rosiglitazone inhibited *MMP-1 *and *MMP-13 *expression in IL-1-β-treated SW-1353 cells at the mRNA and heterogeneous nuclear RNA levels and blunted IL-1-β-induced collagen destruction *in vitro*. Combining LG268 and rosiglitazone had an additive inhibitory effect on *MMP-1 *and *MMP-13 *transcription and collagenolysis. IL-1-β inhibited luciferase expression in the MMP reporter assay, but rosiglitazone and LG268 had no effect. ChIP indicated that treatment with IL-1-β, but not LG268 and rosiglitazone, increased PPARγ at the proximal promoters of both MMPs. Finally, rosiglitazone or LG268 induced 'cross-SUMOylation' of both the target receptor and its binding partner, and IL-1-β-alone had no effect on SUMOylation of RXR and PPARγ but antagonized the ligand-induced SUMOylation of both receptors.

**Conclusions:**

The PPARγ and RXR ligands rosiglitazone and LG268 may act through similar mechanisms, inhibiting *MMP-1 *and *MMP-13 *transcription. Combinatorial treatment activates each partner of the RXR:PPARγ heterodimer and inhibits IL-1-β-induced expression of *MMP-1 *and *MMP-13 *more effectively than either compound alone. We conclude that the efficacy of combined treatment with lower doses of each drug may minimize potential side effects of treatment with these compounds.

## Introduction

The matrix metalloproteinases (MMPs) are a family of zinc-dependent endopeptidases responsible for the degradation of extracellular matrix (ECM) components. While low levels of these enzymes are required for the homeostatic ECM turnover seen in wound healing, angiogenesis, and development, high levels have been implicated in the pathology of atherosclerosis, tumor metastasis, and the arthritides. In the case of osteoarthritis (OA) and rheumatoid arthritis (RA), members of the collagenase subgroup of the MMPs, specifically *MMP-1 *and *MMP-13*, are particularly important in the progression of joint disease [1,2]. The ability to cleave the collagen triple helix is unique to the collagenases, and the overexpression of *MMP-1 *and *MMP-13 *in chondrocytes in response to proinflammatory cytokines such as interleukin-1-beta (IL-1β) and tumor necrosis factor-alpha is critical in the pathogenesis of OA and RA [1].

Many efforts to design small-molecule inhibitors of MMP activity (MMPIs) have succeeded in creating potent compounds; however, due to the highly conserved nature of the catalytic domain among family members, these compounds demonstrate significant inhibitory efficacy against multiple MMPs [3]. This lack of specificity has been identified as the likely cause of the debilitating side effects observed in clinical trials with these compounds which presented as a chronic musculoskeletal syndrome (MSS) that was characterized by reduced mobility with joint pain and edema due to tendonitis and inflammation [4-6]. The root cause of the MSS is thought to be the disruption of normal connective tissue turnover, secondary to the inhibition of multiple MMPs [7]. Unfortunately, the MSS has continued to hinder many newly developed MMPIs, resulting in the discontinuation of multiple clinical trials [8], although promising results with MMP-specific compounds are emerging [9]. Specific inhibition of MMP gene synthesis is an alternative strategy for counteracting the overexpression of MMPs involved in particular diseases. Although many MMP promoters share similarities, particular variations in MMP promoter structure and in the signaling pathways required for their expression may make it possible to target certain family members with specific ligands.

Peroxisome proliferator-activated receptor-gamma (PPARγ) is a nuclear hormone receptor (NHR) initially recognized as a regulator of genes active in adipogenesis and insulin sensitivity [10]. PPARγ forms an obligate heterodimer with the retinoid X receptor (RXR:PPARγ) that binds to direct repeat-1 (DR-1) motifs, known as PPARγ response elements (PPREs), in the promoter DNA of regulated genes [11]. NHRs are typically thought to exert their transcriptional regulatory effects through interaction with coregulatory complexes, which modify the local chromatin environment via multiple mechanisms, including the enzymatic activity of histone deacetylases (HDACs) and histone acetyltransferases (HATs) [12]. HDAC activity results in a decrease in histone acetylation and a subsequent decrease in transcriptional activity, whereas HAT activity leads to an increase in histone acetylation and a subsequent increase in transcriptional activity [13].

Recent work has identified an anti-inflammatory role for PPARγ in chondrocytes when the receptor is activated by ligands such as the thiazolidinedione compound rosiglitazone and the prostaglandin 15-Deoxy-Δ12,14-prostaglandin J2 [2,14-16]. Notably, this anti-inflammatory effect of PPARγ ligands extends to the inhibition of IL-1β-induced expression of *MMP-1 *and *MMP-13 *in rabbit chondrocytes [2,17,18], and administration of these compounds blunts the development of joint disease in animal models of arthritis [18,19]. François and colleagues [17] have proposed a mechanism to explain rosiglitazone-mediated inhibition of IL-1β-induced expression of rabbit *MMP-1 *that involves binding of the RXR:PPARγ heterodimer to a degenerate DR-1 site in the proximal (approximately -72 base pairs) region of the rabbit *MMP-1 *promoter. This DR-1 site overlaps a binding site for the transcription factor activator protein-1 (AP-1), which is largely responsible for the proinflammatory cytokine-induced upregulation of *MMP-1 *[20]. In this competitive binding model, binding of the RXR:PPARγ heterodimer to the DR-1 element precludes binding of AP-1 proteins to its site and thereby antagonizes the expression of *MMP-1*. François and colleagues [17] also identified a similar degenerate DR-1/AP-1 site in the promoters of human *MMP-1, MMP-9*, and *MMP-13*, although the function of this site has not been experimentally verified in the human genes.

Previous work by our laboratory has shown that LG100268 (LG268), a ligand specific for RXR, inhibits IL-1β-induced *MMP-1 *and *MMP-13 *transcription in the SW-1353 human chondrosarcoma cell line and is associated with a decrease in histone acetylation proximal to the transcription start site in the *MMP-1 *and *MMP-13 *promoters [21]. While RXR is an obligate dimer partner for a number of other NHRs, including retinoic acid receptors, thyroid receptor, vitamin D receptor, PPARs, liver X receptors (LXRs), and farnesoid X receptor (FXR) [22], the ligand LG268 activates only a subset of the RXR catalog of partners, including RXR:FXR, RXR:LXR, RXR:PPARα, and RXR:PPARγ heterodimers, as well as RXR homodimers [23-25]. Of these dimers, only RXR:PPARα, RXR:PPARγ, and RXR homodimers bind to the DR-1 element [11], suggesting that all or any of these three dimers may be responsible for mediating the inhibitory effect of LG268 on *MMP-1 *and *MMP-13*. However, since PPARγ-specific, but not PPARα, ligands block *MMP-1 *and *MMP-13 *gene expression, RXR:PPARγ heterodimers as well as RXR homodimers may be mediating this suppression [2,18,26].

Recent investigations into the mechanisms by which PPARγ inhibits the expression of genes involved in inflammation have identified a molecular pathway of ligand-dependent conjugation of the small ubiquitin-like modifier (SUMO) to lysines in the PPARγ receptor [14,27]. This SUMO-conjugated form of PPARγ then binds to corepressor complexes containing HDAC activity and to other promoter-bound proteins. This anchors the corepressors and prevents their release upon proinflammatory stimulation, thereby blocking recruitment of coactivator complexes with HAT activity. The presence of multiple functional SUMOylation sites (SUMO consensus sequence = ψ KXE/D, where ψ is a hydrophobic amino acid, X is any amino acid, and K is the specific SUMOylation target) within PPARγ has been confirmed [14,27], and Floyd and colleagues [27] describe multiply SUMOylated forms of PPARγ. Pascual and colleagues [14] demonstrate that SUMOylation at different sites confers different modifications of receptor activity and identify K365 as the SUMOylation site required for transrepression of inflammatory genes by PPARγ. SUMOylation of RXR has also been reported [28].

We hypothesized that, because LG268 and PPARγ ligands target the same NHR complex and have similar inhibitory effects on MMP production, both ligands may be activating similar mechanisms to inhibit MMP gene expression. The competitive binding model implicates competition for binding to the degenerate DR-1 site between RXR:PPARγ and AP-1 proteins as a possible mechanism for rosiglitazone-mediated inhibition of *MMP-1 *[17]. In addition, we hypothesized that LG268, as a ligand for RXR, may also induce increased binding of the heterodimer to the DR-1 site and that combination treatment with both ligands would further increase binding to the DR-1 site since both NHRs would be liganded. As a result, combined treatment should lead to greater inhibition of *MMP-1 *and *MMP-13 *gene expression compared with either compound alone. In this paper, we demonstrate that combined treatment with the RXR ligand LG268 and the PPARγ ligand rosiglitazone suppresses *MMP-1 *and MMP13 gene expression more effectively than either compound alone. In addition, we document that this inhibition is transcriptionally mediated and involves genetic and epigenetic mechanisms but does not appear to involve competitive binding between RXR:PPARγ and AP-1 at the DR-1/AP-1 element.

## Materials and methods

### Cell culture

SW-1353 human chondrosarcoma cells were obtained from the American Type Culture Collection (Manassas, VA, USA). These cells were propagated at 37°C with 5% CO in Dulbecco's modified Eagle's medium (DMEM) (Mediatech, Inc., Manassas, VA, USA) containing 10% fetal bovine serum (FBS) (HyClone, Logan, UT, USA), 100 U/mL penicillin, 100 μL/mL streptomycin, and 2 mM glutamine. Cells were washed three times with Hanks' balanced salt solution (HBSS) and passaged 1:10 using 0.25% trypsin (Mediatech, Inc.). Experiments were performed with cells from passages 10 to 30, and subsequent cultures were refreshed from frozen stocks.

### Cell treatments

The synthetic rexinoid LG268 was kindly provided by Ligand Pharmaceuticals (San Diego, CA, USA). LG268 and the PPARγ ligands rosiglitazone and GW-9662 were solubilized in dimethylsulfoxide, stored in 10 μM aliquots at -20°C, and added to culture media at varying concentrations. Recombinant human IL-1β (Promega Corporation, Madison, WI, USA) was solubilized in sterile H_2_O, stored in 10 μg/mL aliquots at -80°C, and added to media at 1 ng/mL. For most experiments, SW-1353 cells were grown to approximately 90% confluence in six-well plates and washed twice with HBSS to remove trace serum and waste metabolites. Two milliliters of serum-free DMEM supplemented with 0.2% lactalbumin hydrosylate (DMEM/LH) and appropriate concentrations of LG268 and/or rosiglitazone were added for 1 to 24 hours. IL-1β was then added to the media for an additional 1 to 24 hours followed by cell harvest.

### Quantitative real-time reverse transcription-polymerase chain reaction

After experimental treatment, the cells were washed twice with cold 1× phosphate-buffered saline (PBS), scraped off the plate, and homogenized using QIAshredder spin columns (Qiagen Inc., Valencia, CA, USA). Total cellular RNA was isolated using the RNeasy Mini Kit (Qiagen Inc.) in accordance with the manufacturer's instructions, including DNA contamination removal by on-column treatment with the RNase-Free DNase Kit (Qiagen Inc.). The reverse transcription (RT) reaction was performed on 4 μg of purified total RNA using Moloney murine leukemia virus reverse transcriptase (Invitrogen Corporation, Carlsbad, CA, USA) with oligo(dT) or random hexamer primers (Applied Biosystems, Foster City, CA, USA) for mRNA and heterogeneous nuclear RNA (hnRNA) studies, respectively. The RT reactions were performed in a PTC-100 thermal cycler (MJ Research, now part of Bio-Rad Laboratories, Inc., Hercules, CA, USA). Real-time polymerase chain reaction (PCR) was performed using the SYBR Green PCR Master Mix kit (Applied Biosystems) in accordance with the manufacturer's instructions. PCRs were run with experimental triplicates and machine (on-plate) duplicates or triplicates for each sample. To enable quantitative between-plate comparisons, standard curves were generated with each mRNA assay. Both experimental and standard reactions were run using 125 ng each of the appropriate forward and reverse primers for the MMPs analyzed (sequences described previously in [21]). Target gene expression was normalized to glyceraldehyde 3-phosphate dehydrogenase (GAPDH) mRNA expression and reported as mean copies ± standard deviation of target gene mRNA per copy of GAPDH mRNA. Several real-time RT-PCR experiments in which standard curve plasmids were not available were performed. In these cases, the relative mRNA levels of the experimental gene under different treatment conditions were normalized to GAPDH mRNA levels using the 2^-ΔΔCt ^statistical method [29].

### Western blotting

Trichloracetic acid (TCA) protein precipitation and Western blotting were performed as described previously [21]. Briefly, SW-1353 cells were grown to confluency in six-well plates in DMEM with 10% FBS. The media were aspirated, the cells were washed with HBSS, and 2 mL of DMEM/LH was added to each well. Cells were pretreated for 24 hours with rosiglitazone, LG268, or both, and IL-1β was added for an additional 24 hours. Protein was TCA-precipitated from 1 mL of media from each well and resuspended in 40 mL of Laemmli buffer. Samples were resolved using Tris-HEPES-SDS precast 10% polyacrylamide gels (catalog number 25201; Pierce, Rockford, IL, USA) and transferred to an Immobilon-P polyvinylidene difluoride membrane (Millipore Corporation, Billerica, MA, USA). The membranes were probed for *MMP-1 *using a polyclonal rabbit anti-human *MMP-1 *antibody (AB8105; Chemicon International, Temecula, CA, USA) or for *MMP-13 *with a polyclonal *MMP-13 *antibody generously provided by Peter Mitchell (Pfizer Inc, New York, NY, USA). Protein bands were visualized by incubation with a goat anti-rabbit secondary antibody conjugated to horseradish peroxidase (Cell Signaling Technology, Inc., Danvers, MA, USA) and enhanced chemiluminescence analysis with the Western Lightning reagent (PerkinElmer Life and Analytical Sciences, Inc., Waltham, MA, USA).

### Collagen degradation assay

This assay was performed as previously described [21,30]. Briefly, fibrillar collagen preparations were made from Vitrogen 100 bovine type I collagen (Cohesion Technologies, Inc., Palo Alto, CA, USA) in accordance with the manufacturer's instructions. The collagen solution was diluted to 2 mg/mL and the pH was adjusted to 7.3 ± 0.2. Once neutralized, an equivalent volume of DMEM/LH containing SW-1353 cells was added, resulting in a final collagen concentration of 1 mg/mL and 2.5 × 10^5 ^cells per well of a six-well plate. Rosiglitazone, LG268, or both were added to the collagen/cell suspension of specific experimental wells. Following incubation at 37°C for 60 minutes, the collagen gelled and 1 mL of DMEM/LH was added on top of the cell-containing collagen plug. After 24 hours of incubation in DMEM/LH, IL-1β was added to the media to induce MMP production and subsequent collagen degradation. Approximately 24 hours after the addition of IL-1β, the media were removed from each well and weighed to quantify the extent of collagen degradation.

### Luciferase reporter assays

Luciferase reporter plasmids incorporating four copies of the putative overlapping DR-1/AP-1 site of the *MMP-1 *(MMP1-ENDOG-Luc) or *MMP-13 *(MMP13-ENDOG-Luc) promoters were constructed using the pGL3-basic plasmid (Promega Corporation). Control reporters were constructed in a similar fashion, with four scrambled copies of the DR-1/AP-1 element of *MMP-1 *(MMP1-SCRAM-Luc) or *MMP-13 *(MMP13-SCRAM-Luc). SW-1353 cells were plated in six-well plates at a density of 1.5 × 10^5 ^cells per well. The next day, cells were transiently transfected in six-well plates with 2 μg/well of the PPRE-tk-luciferase plasmid [31], or the custom DR-1/AP-1-luciferase plasmids described above, using 5 μL/well of Lipofectamine 2000 (Invitrogen Corporation) in accordance with the manufacturer's instructions. Four to six hours after transfection, cells were washed twice with HBSS followed by the addition of 2 mL of DMEM/LH media containing the indicated NHR ligand. After 24 hours of ligand treatment, IL-1β was added to the media for an additional 24 hours. The cells were then washed three times with cold 1× PBS, and lysates were harvested using 1× Passive Lysis Buffer (Promega Corporation). Protein concentration was determined using Bio-Rad Protein Assay reagent (Bio-Rad Laboratories, Inc.), and equal amounts of total protein were loaded for each sample. Luciferase activity was measured in relative light units using an Lmax II luminometer (Molecular Devices Corporation, Sunnyvale, CA, USA).

### Chromatin immunoprecipitation

The chromatin immunoprecipitation (ChIP) protocol was adapted from the 'fast ChIP method' [32]. SW-1353 cells were grown to confluence in 150-mm plates (approximately 10^7 ^cells). Crosslinking was performed by adding 40 μL of 37% formaldehyde per milliliter of cell culture media directly to the culture media, and the plates were rocked gently at room temperature for 10 minutes. Crosslinking was quenched by adding 141 μL of 1 M glycine per milliliter media and gently rocking for 5 minutes at room temperature. Cells were washed twice with ice-cold 1 × PBS, scraped, and collected in 15-mL conical tubes on ice. Cells were pelleted by centrifugation at 2,000 *g *for 5 minutes at 4°C, resuspended in 1 mL of ChIP buffer (150 mM NaCl, 50 mM Tris HCl pH 7.5, 5 mM EDTA [ethylenediaminetetraacetic acid], 0.5% NP40, 1% Triton X-100) with protease inhibitors (complete mini tabs; Roche, Nutley NJ, USA), and lysed on ice for 10 minutes. Nuclei were collected by centrifugation at 12,000 *g *for 1 minute at 4°C and then washed twice by aspirating the supernatant and resuspending with 1 mL of ChIP buffer. Chromatin was sonicated on ice with 15 × 15 second pulses at power setting #40 on a Sonics Vibro-Cell VC 130PB-1 ultrasonoic processor (Newtown, CT. USA). Debris was cleared by centrifugation at 12,000 *g *for 10 minutes at 4°C, and the supernatant was split into 200-μL aliquots in 1.5-mL microcentrifuge tubes for immunoprecipitation (IP). Two micrograms of specific antibodies to the HA epitope tag (Abcam, Cambridge, UK), acetylated histone H4 (Upstate, now part of Millipore Corporation), PPARγ (Santa Cruz Biotechnology, Inc., Santa Cruz, CA, USA), or normal IgG was added to each tube, and tubes were rotated overnight at 4°C. Twenty microliters of protein A/G agarose (Santa Cruz Biotechnology, Inc.) per IP was washed three times, resuspended 1:1 with ChIP buffer, and distributed (40 μL per IP) to 1.5-mL microcentrifuge tubes. IP reactions were centrifuged at 12,000 *g *for 10 minutes at 4°C, and then 180 μL of supernatant was transferred to the protein A/G agarose tubes and rotated for 45 minutes at 4°C. Beads were collected by centrifugation at 2,000 *g *for 30 seconds at 4°C and then washed five times by removing the supernatant and resuspending in ice-cold ChIP buffer. After washing, the pellet was resuspended in 100 μL of 10% Chelex-100 (Fisher Scientific Co., Pittsburgh, PA, USA), boiled for 10 minutes, and then cooled on ice. One microliter of proteinase K (20 μg/μL) was added to the cooled solution, vortexed, incubated at 55°C for 30 minutes, boiled for 10 minutes, and centrifuged at 12,000 *g *for 1 minute. Eighty microliters of supernatant was transferred to a new microcentrifuge tube, and 120 μL of water was added back to the original tube, vortexed, and centrifuged as before, and 120 μL of supernatant was transferred to the previous 80 μL. Samples were stored at -20°C or immediately quantified using real-time PCR with primers flanking the DR-1/AP-1 site or with negative-control primers flanking an upstream control region (-3 kb for *MMP-1 *and -1 kb for *MMP-13*), normalized to IgG-precipitated DNA, and expressed as a fold-change over untreated cells.

### Immunoprecipitation

For the SUMO IP experiments, cellular proteins were immunoprecipitated following the ExactaCruz system instructions from Santa Cruz Biotechnology, Inc. Briefly, cells were grown to confluency in 150-mm dishes and treated for 1 hour with LG268 (50 nM) and/or rosiglitazone (50 nM) in serum-free DMEM/LH media. The cells were then treated with 1 ng/mL IL-1β for 1 hour and harvested using cold radioimmunoprecipitation assay buffer. Cell lysates were homogenized using QIAshredder columns. IP reactions were performed using 4 μg of anti-SUMO-1 (Santa Cruz Biotechnology, Inc.). IP fractions were resolved using PAGE as described above. The presence of RXR and PPARγ in the IP fractions was detected using 4 μg of RXR (ΔN197) or PPARγ (H-100) antibodies from Santa Cruz Biotechnology, Inc.

## Results

### Rosiglitazone inhibits *MMP-1 *and *MMP-13 *gene expression in chondrocytic cells

**Figure 1:**
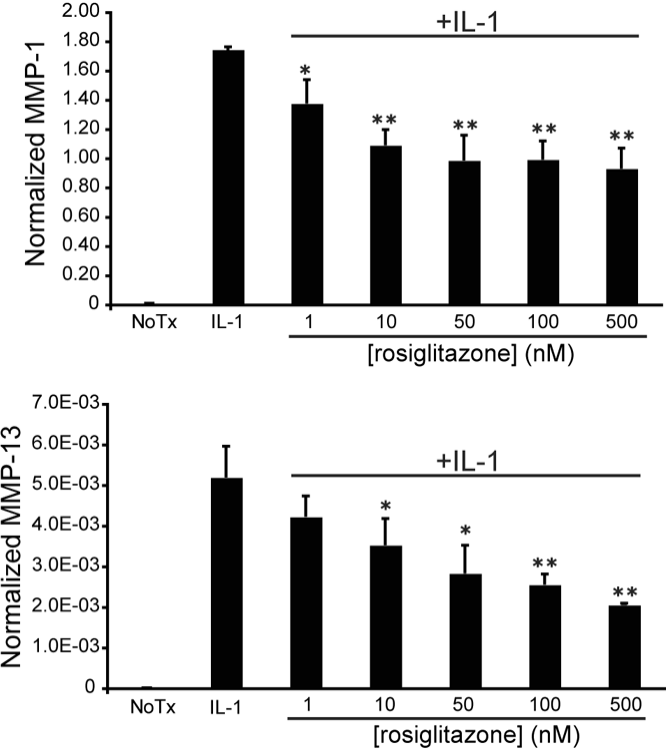
Rosiglitazone inhibits matrix metalloproteinase-1 (*MMP-1*) and *MMP-13 *mRNA production in a dose-dependent manner. SW-1353 cells were treated with varying concentrations of rosiglitazone for 24 hours followed by 24 hours of treatment with 1 ng/mL interleukin-1-beta (IL-1β). Total RNA was harvested, and *MMP-1 *and *MMP-13 *mRNA levels were quantified using real-time reverse transcription-polymerase chain reaction. Y values are given as molecules of MMP per molecule of GAPDH (glyceraldehyde 3-phosphate dehydrogenase) mRNA. There is no statistical difference between *MMP-13 *mRNA levels at concentrations of 50 and 500 nM (*P *= 0.16). *P *values were calculated for the difference from the IL-1β sample using the Student *t *test (*P < 0.05, **P < 0.005). NoTx, no treatment; Rosi, rosiglitazone.

The SW-1353 chondrosarcoma cell line is a model for inflammatory cytokine-induced protease production by human chondrocytes [20,33,34], and we used these cells to quantify the effects of rosiglitazone treatment on IL-1β-stimulated levels of *MMP-1 *and *MMP-13 *mRNA. Cells were incubated for 24 hours in serum-free media containing varying doses of rosiglitazone, followed by IL-1β treatment for 24 hours. Previously, we determined that simultaneous treatment with IL-1β and LG268 leads to modest but significant inhibition of *MMP-1 *and *MMP-13*, and a 12- to 24-hour pretreatment is necessary for maximum inhibition of MMP levels in these cells by the RXR ligand, LG268 [21]. The need for pretreatment is consistent with the mechanism involving SUMOylation-dependent anchoring of PPARγ and associated corepressor complexes at a target gene promoter, whereby pretreatment with rosiglitazone prevents the clearance of corepressor complexes by inflammatory stimuli ([14] and see Discussion). Real-time RT-PCR was used to quantify MMP mRNA, and Figure 1 demonstrates a dose-dependent inhibition of *MMP-1 *and *MMP-13 *mRNA levels in response to rosiglitazone treatment. Significant inhibition of IL-1β-induced *MMP-1 *and *MMP-13 *is seen with 10 nM rosiglitazone, with maximal inhibition at approximately 50 nM. The maximum expression of *MMP-1 *and *MMP-13 *mRNA with rosiglitazone treatment is approximately 50% of that seen with IL-1β alone, paralleling results obtained with LG268 [21].

**Figure 2:**
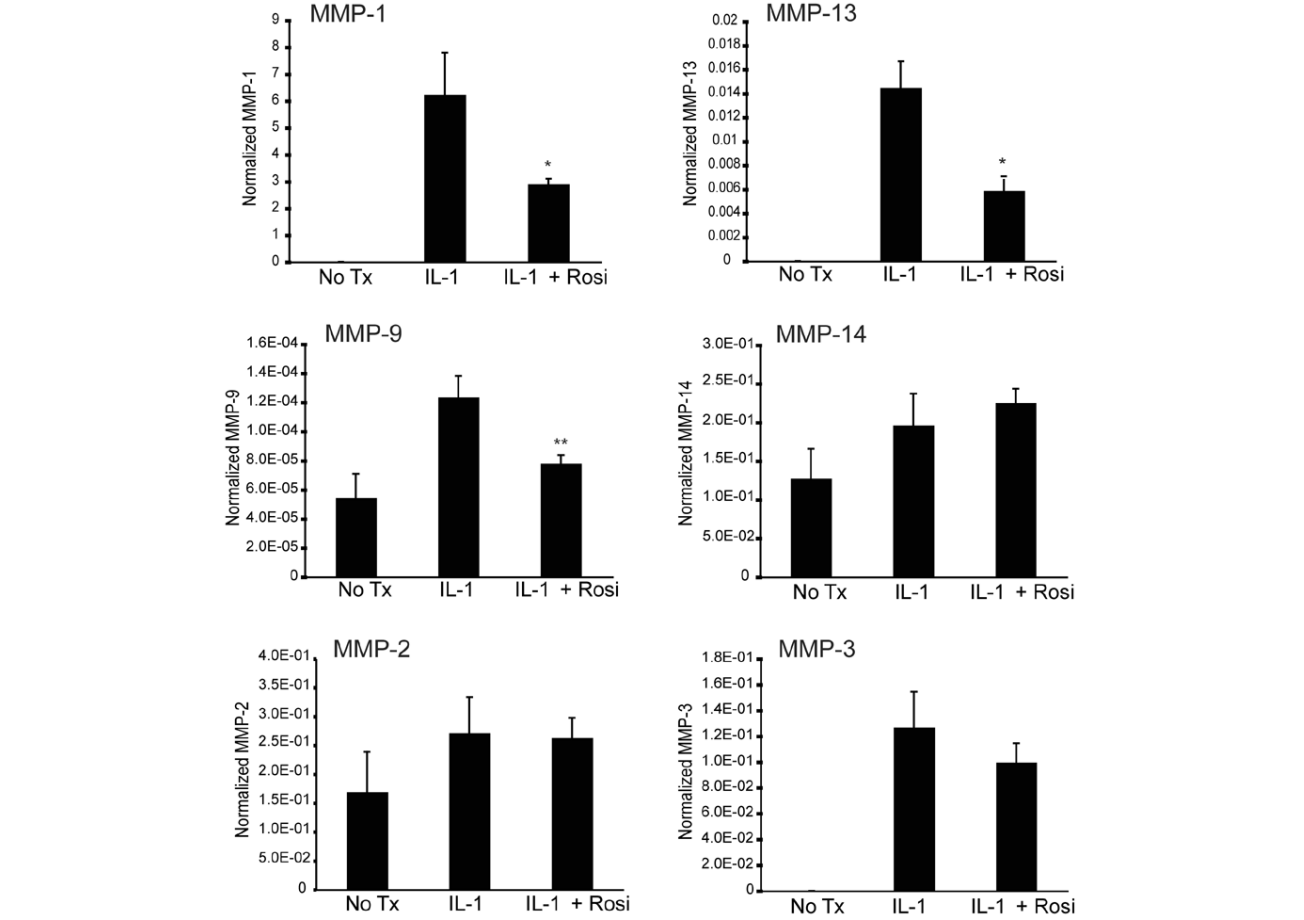
Matrix metalloproteinase-1 (*MMP-1*) and *MMP-13 *mRNA production is specifically inhibited by rosiglitazone treatment. SW-1353 cells were incubated with 50 nM rosiglitazone for 24 hours followed by 1 ng/mL interleukin-1-beta (IL-1β) treatment for an additional 24 hours. Total RNA was harvested, and MMP mRNA levels were quantified using real-time reverse transcription-polymerase chain reaction. Y values are given as molecules of MMP per molecule of GAPDH (glyceraldehyde 3-phosphate dehydrogenase) mRNA. *P *values were calculated for the difference from the IL-1β sample using the Student *t *test (*P < 0.05, **P < 0.005). NoTx, no treatment; Rosi, rosiglitazone.

Next, we investigated the specificity of rosiglitazone inhibition against a panel of MMPs. This panel included MMPs that are responsive to IL-1β stimulation (*MMP-1, MMP-3*, *MMP-9*, and *MMP-13*) as well as those that are constitutively expressed in these cells (MMP-2 and MMP-14). Cells were treated with 50 nM rosiglitazone for 24 hours before stimulation with IL-1β. Using real-time RT-PCR analysis, we observed that rosiglitazone treatment significantly inhibited IL-1β induction of *MMP-1 *and *MMP-13 *while having either a very modest effect (*MMP-9*) or no effect (*MMP-2*, *MMP-3*, and *MMP-14*) on other MMP family members (Figure 2). With rosiglitazone, both the maximum level of inhibition of *MMP-1 *and *MMP-13 *(50% to 60%) and the pattern of MMP inhibition mirror those previously seen with LG268 treatment [21], suggesting that these compounds may be acting through similar mechanisms.

### Combined treatment with rosiglitazone and LG268 further reduces *MMP-1 *and *MMP-13 *mRNA and hnRNA

**Figure 3:**
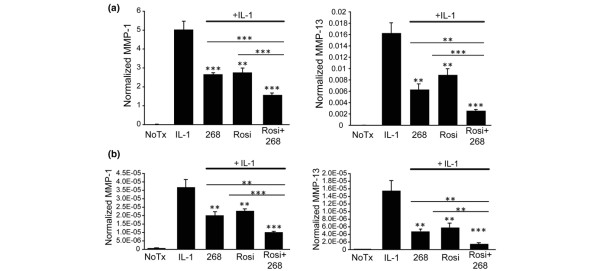
Combination treatment with LG268 and rosiglitazone results in increased inhibition of matrix metalloproteinase-1 (*MMP-1*) and *MMP-13 *expression. SW-1353 cells were treated for 24 hours with 50 nM LG268, 50 nM rosiglitazone, or both compounds followed by 24 hours of treatment with 1 ng/mL interleukin-1-beta (IL-1β). Total RNA was harvested, and MMP **(a) **mRNA and **(b) **heterogeneous nuclear RNA levels were quantified using real-time reverse transcription-polymerase chain reaction. Y values are given as molecules of MMP per molecule of GAPDH (glyceraldehyde 3-phosphate dehydrogenase). *P *values above each vertical bar were determined for the difference from the IL-1β sample, and *P *values above the horizontal bars were determined for the difference between samples on either end of the bar. In all cases, *P *values were calculated using the Student *t *test (**P < 0.005, ***P < 0.0005). 268, LG100268; NoTx, no treatment; Rosi, rosiglitazone.

Considering the parallel effects on MMP production with rosiglitazone and LG268 treatment and the fact that these ligands share a molecular target (RXR:PPARγ heterodimers), we measured the effects on IL-1β-induced *MMP-1 *and *MMP-13 *levels when the two ligands were added in combination. SW-1353 cells were treated for 24 hours with 50 nM LG268, 50 nM rosiglitazone, or the combination of both treatments followed by IL-1β stimulation for 24 hours. As shown in Figure 3a, treatment with either LG268 or rosiglitazone effectively reduced *MMP-1 *and *MMP-13 *mRNA by approximately 50%, and treatment with both ligands led to significantly greater inhibition (approximately 75%) than either drug alone. This is consistent with the idea that treatment with both ligands might increase the binding of RXR:PPARγ to the DR-1 elements in the *MMP-1 *and *MMP-13 *promoters, thereby displacing AP-1 transcription factors and causing greater inhibition of mRNA production.

To determine whether the inhibitory effects of rosiglitazone and the combination treatment with LG268 were due, at least in part, to effects on the rate of transcription, we performed real-time RT-PCR analysis of *MMP-1 *and *MMP-13 *hnRNA levels [21,34,35]. Similar to the mRNA results, treatment with LG268 or rosiglitazone alone resulted in equivalent decreases in IL-1β-stimulated *MMP-1 *and *MMP-13 *hnRNA levels (Figure 3b), indicating an effect at the transcriptional level. With combined treatment, hnRNA levels for both *MMP-1 *and *MMP-13 *were significantly lower when compared with cells treated with a single compound, paralleling the effects seen on mRNA. This inhibition with combined treatment appears to be additive, again suggesting that the compounds may be acting through similar mechanisms.

### Rosiglitazone and LG268 inhibit *MMP-1 *and *MMP-13 *protein production and collagen destruction by SW-1353 cells

**Figure 4:**
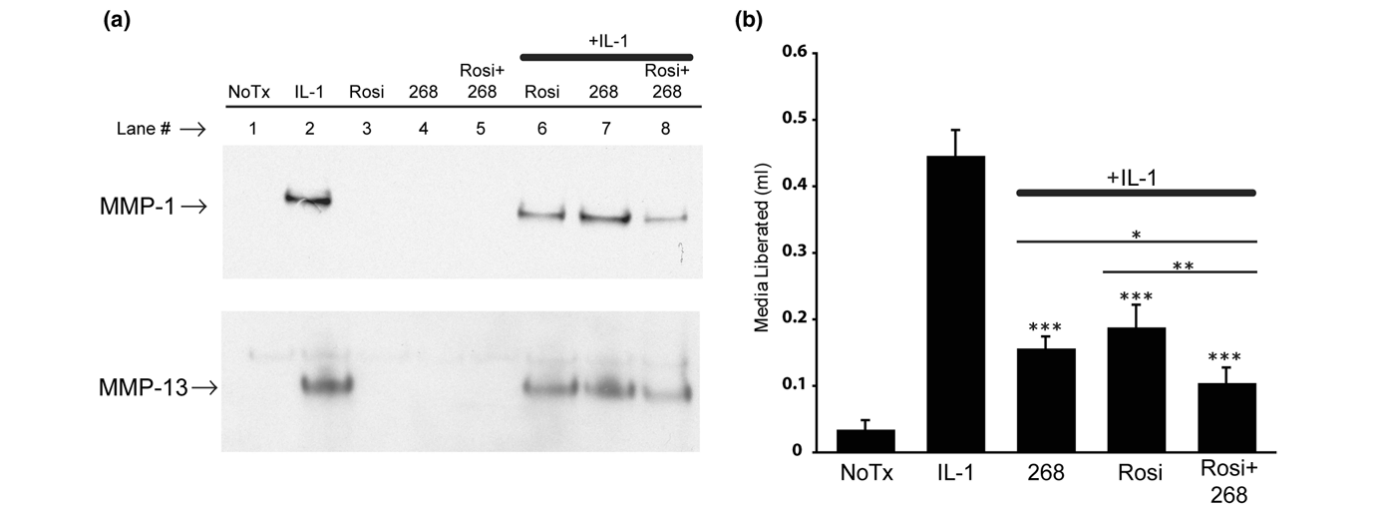
Protein levels and collagenolytic activity more strongly inhibited by dual treatment with LG268 and rosiglitazone. **(a) **SW-1353 cells were pretreated for 24 hours in serum-free media with 50 nM LG268, 50 nM rosiglitazone, or both, followed by treatment with interleukin-1-beta (IL-1β) for 24 hours. Protein was trichloracetic acid-precipitated from 1 mL of the conditioned media and resuspended in 40 μL of Laemmli buffer, and the entire sample was resolved using Tris-HEPES-SDS-PAGE and then transferred to a polyvinylidene difluoride membrane that was probed with anti-*MMP-1 *or anti-*MMP-13 *antibodies. **(b) **SW-1353 cells were embedded in a type I collagen matrix diluted to 1 mg/mL with serum-free media containing 50 nM LG268, 50 nM rosiglitazone, or both compounds. After gelation of the collagen, an additional 1 mL of serum-free media containing 50 nM LG268, 50 nM rosiglitazone, or both compounds was added on top of the gelled collagen and allowed to incubate for 24 hours. IL-1β was then added to the media to stimulate MMP production, and after 24 hours the media was recovered and quantified. Collagen breakdown is indicated by media quantities over 1 g, with the additional media being released from the collagen gel during destruction. Y values are the amount of media recovered over 1 mL. *P *values were calculated for the difference from the IL-1β-treated sample using the Student *t *test (*P < 0.05, **P < 0.005, ***P < 0.0005). 268, LG100268; MMP, matrix metalloproteinase; NoTx, no treatment; Rosi, rosiglitazone.

Figure 3 shows a decrease in expression of *MMP-1 *and *MMP-13 *at the transcriptional level in cells treated with LG268 and rosiglitazone. To determine whether this inhibition extended to the level of MMP protein production and enzymatic activity, we performed Western blot analysis of *MMP-1 *and *MMP-13 *protein levels in conditioned media and an *in vitro *collagen destruction assay looking at the breakdown of type I collagen matrix by IL-1β-stimulated SW-1353 cells [30,36]. A marked increase in *MMP-1 *and *MMP-13 *protein was detected in IL-1β-treated cells (Figure 4a, lane 2). Pretreatment with rosiglitazone or LG268 reduced the amount of *MMP-1 *and *MMP-13 *protein detected (Figure 4a, lanes 6 and 7), and combined pretreatment with rosiglitazone and LG268 together had a greater effect than either compound alone (Figure 4a, lane 8). In the collagen destruction assay, after treatment with LG268 and rosiglitazone either alone or together for 24 hours, IL-1β was added for an additional 24 hours and liberated culture media that had been trapped within the collagen matrix were harvested and quantified by weighing [30,36]. Figure 4b shows that treating the cells with IL-1β resulted in substantial destruction of the collagen matrix, as indicated by the liberation of medium trapped within the matrix. The figure also shows that either LG268 or rosiglitazone decreased collagen destruction by IL-1β-stimulated SW-1353 cells by approximately 50% to 60%. When the drugs were added together, there was even less collagen breakdown than that seen with a single compound, resulting in only 20% of the matrix degradation seen with IL-1β alone. This finding indicates that dual treatment with rexinoids and PPARγ ligands may be an attractive avenue of investigation for the therapeutic inhibition of collagen destruction in arthritis (see Discussion).

### Rosiglitazone and LG268 transactivate a PPRE

**Figure 5:**
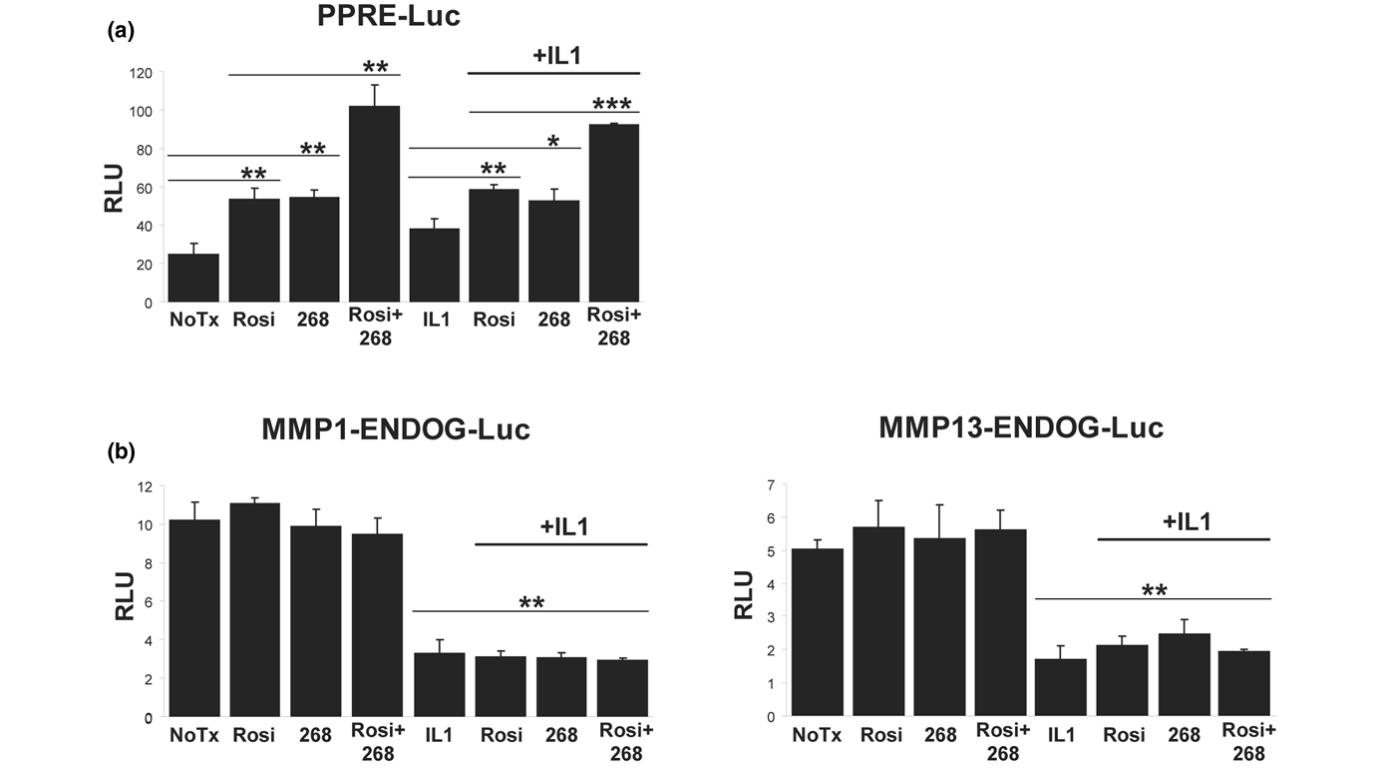
Rosiglitazone and LG268 activate a consensus PPRE-luciferase reporter but not the matrix metalloproteinase (MMP) direct repeat-1/activator protein-1 (DR-1/AP-1) reporters. SW-1353 cells were seeded in six-well plates and transfected with 2 μg/well of the **(a) **PPRE-Luc, **(b) **MMP1-ENDOG-Luc, or MMP13-ENDOG-Luc (see Materials and methods) luciferase reporter constructs and then treated for 24 hours with 50 nM LG268, 50 nM rosiglitazone, or both drugs together, followed by no treatment or 1 ng/mL interleukin-1-beta (IL-1β) for 24 hours. Cells were solubilized in passive lysis buffer, and equal amounts of protein were loaded for each sample and assayed for luciferase activity as reported in relative light units (RLU). Error bars represent standard deviations of biological triplicates. *P *values were calculated using the Student *t *test (*P < 0.05, **P < 0.005, ***P < 0.0005). In (a), there was no statistical difference (*P *> 0.2) between the nuclear receptor ligand-treated samples and their corresponding IL-1β-treated counterparts (for example, rosiglitazone versus rosiglitazone + IL-1β). In (b), *P *values represent the IL-1β-treated group versus the non-IL-1β-treated group. 268, LG100268; NoTx, no treatment; PPRE, peroxisome proliferator-activated receptor-gamma response element; Rosi, rosiglitazone.

The previous figures show that LG268 and rosiglitazone have an inhibitory effect on both the production and activity of *MMP-1 *and *MMP-13 *in IL-1β-stimulated chondrocytic cells. We next wanted to investigate the possible mechanisms behind this inhibition. RXR:PPARγ heterodimers can regulate gene expression through binding to PPRE/DR-1 sites in the promoters of target genes [37]. Therefore, to determine whether RXR:PPARγ heterodimers function as expected in the SW-1353 cell line, we used a luciferase reporter assay to test the response of a canonical PPRE/DR-1 element to treatment with rosiglitazone and LG268. We obtained a luciferase reporter construct, driven by three copies of the consensus PPRE from the rat acyl-CoA oxidase promoter, which is known to be activated by treatment with PPARγ and RXR ligands [31]. SW-1353 cells were transfected with the reporter construct and then treated with LG268 and rosiglitazone either alone or together for 24 hours. The cells were then treated with IL-1β for an additional 24 hours and cell lysates were assayed for luciferase activity. Figure 5a demonstrates that treatment with either LG268 or rosiglitazone led to an approximately twofold increase in luciferase levels as compared with untreated cells, and treatment with both drugs led to even greater activation, approximately fourfold over untreated cells. The addition of IL-1β appeared to have minimal effect on reporter expression. These findings support the conclusions that (a) LG268 and rosiglitazone are each able to activate a consensus PPRE/DR-1 element in the SW-1353 cells and (b) combination treatment leads to synergistic activation of this element, presumably because both partners of the RXR:PPARγ heterodimer are liganded/activated.

### Rosiglitazone and LG268 fail to transactivate the DR-1/AP-1 element

After demonstrating that a canonical PPRE/DR-1 reporter construct responded as expected, we reasoned that if RXR:PPARγ were binding to the DR-1/AP-1 site in the *MMP-1 *and *MMP-13 *proximal promoters, this DNA element may also be responsive to treatment with combinations of LG268 and rosiglitazone. We used a luciferase reporter assay with a construct driven by four copies of the endogenous DR-1/AP-1 element from either the *MMP-1 *or *MMP-13 *promoter. Figure 5b shows that neither the *MMP-1 *(MMP1-ENDOG-Luc) nor the *MMP-13 *(MMP13-ENDOG-Luc) construct was responsive to treatment with rosiglitazone or LG268. The figure also shows that treatment with IL-1β reduced expression of both constructs, which does not reflect the response of endogenous *MMP-1 *and *MMP-13*, whose expression is induced by IL-1β (Figures 1 and 3). Previous studies have shown that reporter gene expression driven by components of the *MMP-1 *and *MMP-13 *promoters often do not mirror expression of the endogenous genes [20,21]. Although Figure 5 suggests that the putative DR-1 element of the DR-1/AP-1 site does not function as a traditional response element for RXR:PPARγ in these cells, the paradoxical response to IL-1β led us to abandon further studies with the DR-1/AP-1 luciferase constructs in favor of a more direct, *in vivo *approach, illustrated by the ChIP studies (see below). Taken together, the luciferase reporter data suggest that mechanisms requiring native chromatin conformation are involved in regulating expression of these genes, including histone modification ([21] and see below) and interaction with factors at other promoter elements [20,21].

### Treatment with IL-1β, but not rosiglitazone or LG268, correlates with an increase in PPARγ at the DR-1/AP-1 site

**Figure 6:**
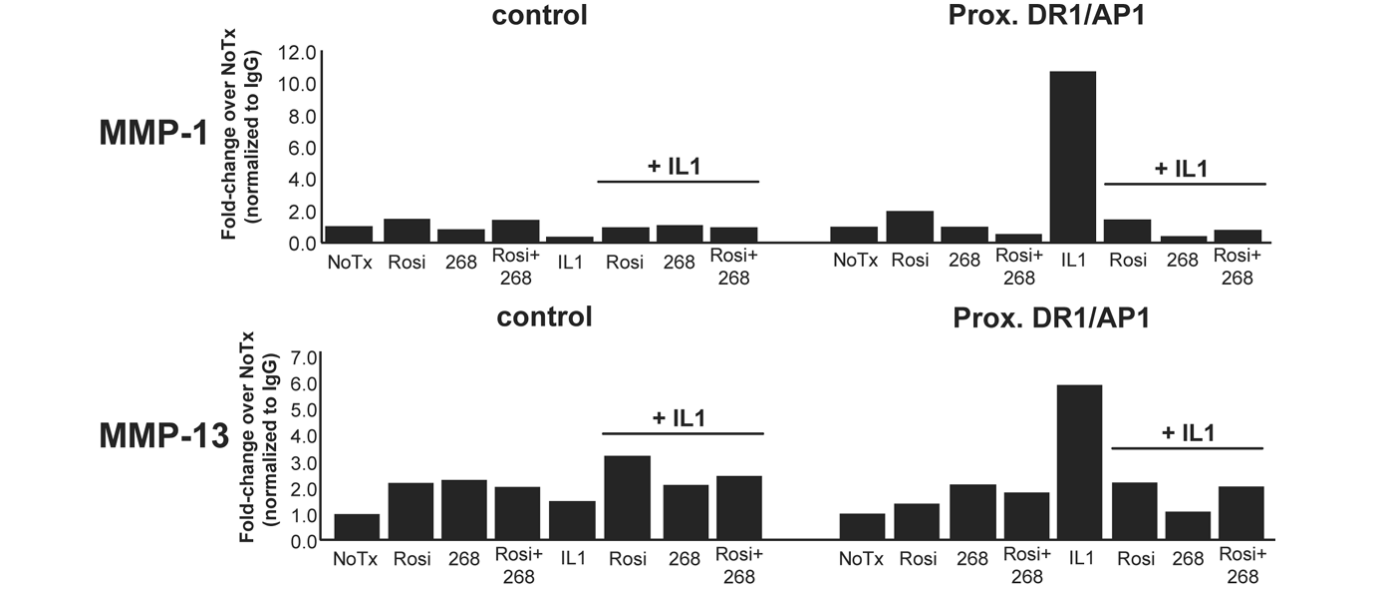
Interleukin-1-beta (IL-1β), but not rosiglitazone or LG268, increases peroxisome proliferator-activated receptor-gamma (PPARγ) at the matrix metalloproteinase-1 (*MMP-1*) and *MMP-13 *direct repeat-1/activator protein-1 (DR-1/AP-1) site. SW-1353 cells were treated for 24 hours with 50 nM LG268, 50 nM rosiglitazone, or both, followed by no treatment or 1 ng/mL IL-1β for 24 hours. Cells were crosslinked with formaldehyde, and nuclei were collected and sonicated to shear chromatin to an average length of 500 base pairs. The crosslinked sonicated chromatin was immunoprecipitated overnight with an antibody to PPARγ and pulled down with protein A/G agarose beads. The immunoprecipitated DNA was treated with Chelex 100 beads followed by proteinase K and used in real-time polymerase chain reaction with primers flanking the DR-1/AP-1 site of *MMP-1 *or *MMP-13 *or with negative-control primers flanking a region of DNA -3 kb upstream from the DR-1/AP-1 in *MMP-1 *or -1 kb upstream for *MMP-13*. Data were normalized to nonspecific IgG-precipitated DNA and expressed as fold-change over untreated cells. Results are representative of at least three independent experiments. 268, LG100268; NoTx, no treatment; Rosi, rosiglitazone.

**Figure 7:**
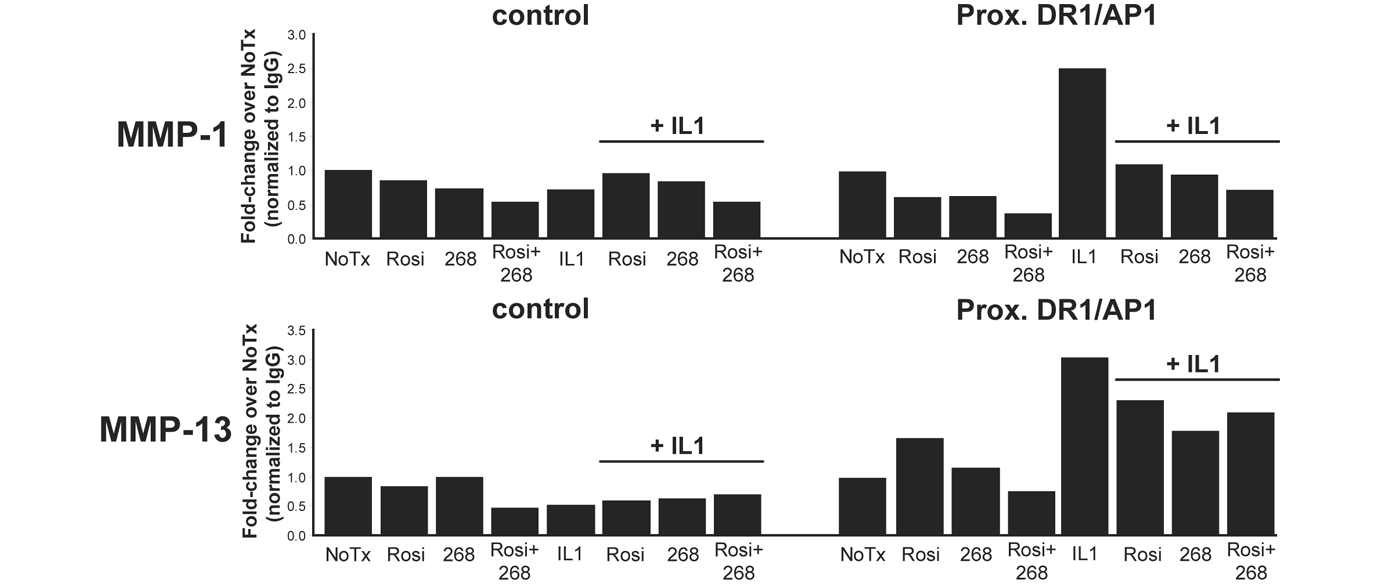
Hemagglutinin-tagged peroxisome proliferator-activated receptor-gamma (HA-PPARγ) chromatin immunoprecipitation (ChIP). SW-1353 cells were transiently transfected with a construct expressing HA-PPARγ and treated for 24 hours with 50 nM LG268, 50 nM rosiglitazone, or both, followed by no treatment or 1 ng/mL interleukin-1-beta (IL-1β) for 24 hours. Cells were crosslinked with formaldehyde, and nuclei were collected and sonicated to shear chromatin to an average length of 500 base pairs. The crosslinked sonicated chromatin was immunoprecipitated overnight with an antibody to the hemagglutinin tag and pulled down with protein A/G agarose beads. The immunoprecipitated DNA was treated with Chelex 100 beads followed by proteinase K and used in real-time polymerase chain reaction with primers flanking the direct repeat-1/activator protein-1 (DR-1/AP-1) site of matrix metalloproteinase-1 (*MMP-1*) or *MMP-13 *or with negative-control primers flanking a region of DNA -3 kb upstream from the DR-1/AP-1 in *MMP-1 *or -1 kb upstream for *MMP-13*. Data were normalized to nonspecific IgG-precipitated DNA and expressed as fold-change over untreated cells. Results are representative of at least three independent experiments. 268, LG100268; NoTx, no treatment; PPARγ, peroxisome proliferator-activated receptor-gamma; Rosi, rosiglitazone.

If LG268 and rosiglitazone increase the affinity of RXR:PPARγ binding to the DR-1/AP-1 site, thereby interfering with binding to that site by the AP-1 transcription factors, then elevated levels of PPARγ would be expected at the DR-1/AP-1 site in cells treated with LG268 and rosiglitazone when compared with cells treated with IL-1β alone. In that regard, we used ChIP assays to test whether endogenous PPARγ was detectable at the DR-1/AP-1 sites in the *MMP-1 *and *MMP-13 *promoters. SW-1353 cells were treated with rosiglitazone, LG268, or both, with or without IL-1β. Sonicated chromatin was immunoprecipitated with anti-PPARγ antibody (catalog number sc-7196 X; Santa Cruz Biotechnology, Inc.) and the enriched DNA was quantified with real-time PCR using primers targeting the DR-1/AP-1 sites of *MMP-1 *and *MMP-13 *or a nonspecific upstream region of the promoter as a negative control (see Materials and methods). The data in Figure 6 are representative of at least three independent experiments. We detected a marked increase in PPARγ at the DR-1/AP-1 site at both promoters in cells treated with IL-1β, which appeared to be blocked by ligand treatment at the *MMP-1 *promoter but only modestly inhibited at the *MMP-13 *promoter (Figure 6). We saw little effect when rosiglitazone or LG268 was added alone. For all conditions, there was little variation at the upstream control region, demonstrating localization of changes in PPARγ binding at the target site. We also performed these experiments in SW-1353 cells transiently transfected with a plasmid expressing hemagglutinin-tagged PPARγ (HA-PPARγ). Endogenous *MMP-1 *and *MMP-13 *mRNA expression was unaffected by the overexpression of HA-PPARγ and responded as seen previously to rosiglitazone, LG268, and IL-1β, as measured by real-time RT-PCR (data not shown). We immunoprecipitated with an antibody to the HA tag and saw similar results (Figure 7). The unexpected increase in PPARγ with IL-1β treatment may suggest a potential role for PPARγ in IL-1β signaling at the DR-1/AP-1 element in the *MMP-1 *and *MMP-13 *promoters (see Discussion). We concluded that these findings do not support the competitive binding model, in which one would expect to see an increase in PPARγ at the DR-1/AP-1 site with rosiglitazone or LG268 treatment as compared with treatment with IL-1β alone.

### IL-1β-induced histone acetylation is inhibited by rosiglitazone and LG268

**Figure 8:**
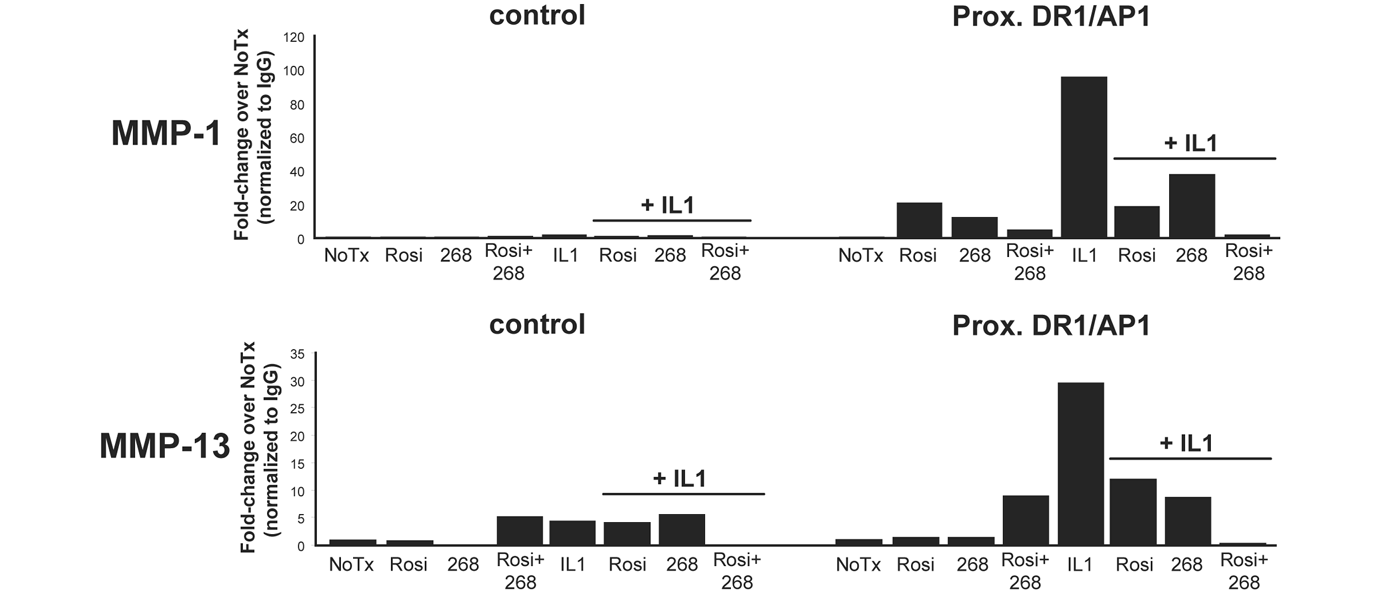
Rosiglitazone and LG268 block interleukin-1-beta (IL-1β)-induced histone acetylation at the direct repeat-1/activator protein-1 (DR-1/AP-1) site. SW-1353 cells were treated for 24 hours with 50 nM LG268, 50 nM rosiglitazone, or both, followed by no treatment or 1 ng/mL IL-1β for 24 hours. Cells were crosslinked with formaldehyde, and nuclei were collected and sonicated to shear chromatin to an average length of 500 base pairs. The crosslinked sonicated chromatin was immunoprecipitated overnight with an antibody to acetylated histone H4 and pulled down with protein A/G agarose beads. The immunoprecipitated DNA was treated with Chelex 100 beads followed by proteinase K and used in real-time polymerase chain reaction with primers flanking the DR-1/AP-1 site of matrix metalloproteinase-1 (*MMP_1*) or *MMP-13 *or with negative-control primers flanking a region of DNA -3 kb upstream from the DR-1/AP-1 in *MMP-1 *or -1 kb upstream for *MMP-13*. Data were normalized to nonspecific IgG-precipitated DNA and expressed as fold-change over untreated cells. Results are representative of at least three independent experiments. 268, LG100268; NoTx, no treatment; Rosi, rosiglitazone.

RXR:PPARγ is known to affect the transcription of target genes via interaction with coactivator and corepressor complexes that modify histones in the target gene promoter by acetylating and deacetylating core histone subunits, including histone subunit H4 [12]. LG268 has been shown to prevent histone acetylation at the proximal promoter region of both *MMP-1 *and *MMP-13 *in IL-1β-treated SW-1353 cells [21]. We used ChIP assays, as described above, with antibodies to acetylated histone H4 to detect changes in acetylation of histones at the DR-1/AP-1 element in both *MMP-1 *and *MMP-13 *promoters in SW-1353 cells treated with rosiglitazone, LG268, or both, with or without IL-1β. At the DR-1/AP-1 element in both promoters, IL-1β treatment led to a marked increase in histone acetylation (Figure 8), consistent with HAT recruitment, H4 acetylation, and subsequent transcriptional activation [13]. This increase in acetylation was blocked by treatment with either rosiglitazone or LG268, consistent with the recruitment of HDACs and subsequent transcriptional repression [13]. Importantly, combined treatment with rosiglitazone and LG268 led to a dramatic decrease in H4 acetylation at both the *MMP-1 *and *MMP-13 *promoters, suggesting that decreased acetylation may be a prominent mechanism by which these two ligands decrease transcriptional activity of these genes (see Discussion). We also note that, as seen previously in Figure 6, there was little variation at the upstream control region, demonstrating localization of alterations in H4 acetylation to the DR-1/AP-1 site. These data, considered with the PPARγ ChIP results (Figures 6 and 7), suggest that rosiglitazone and LG268 may be inhibiting the IL-1β-induced transcription of *MMP-1 *and *MMP-13 *not by a physical blockade of factor binding but through a mechanism involving interaction with HDAC-containing coregulatory complexes and regulation of histone acetylation [12].

### Treatment with rosiglitazone and LG268 leads to SUMOylation of PPARγ and RXR

**Figure 9:**
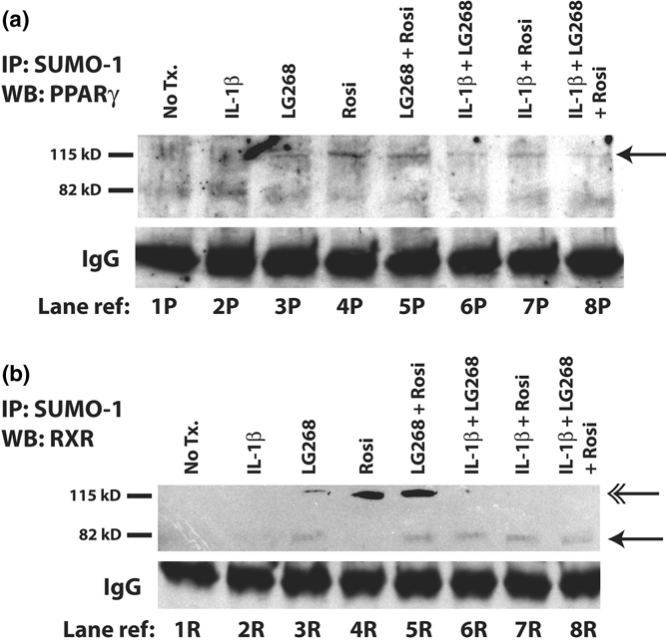
LG268 and rosiglitazone induce SUMOylation of their respective receptors and the unliganded partner. LG268 and rosiglitazone treatment increases SUMOylation of **(a) **peroxisome proliferator-activated receptor-gamma (PPARγ) and **(b) **retinoid X receptor (RXR). SW-1353 cells were treated with 1 hour of 50 nM LG268, 50 nM rosiglitazone, or both for pretreatment and then 1 hour of 1 ng/mL interleukin-1-beta (IL-1β) for stimulation. Cells were harvested in 1× radioimmunoprecipitation assay lysis buffer, and total protein was quantified. Two thousand micrograms of total cell protein and 4 μg of anti-SUMO1 antibody were used in each immunoprecipitation reaction. Immunoprecipitated proteins were resolved using PAGE; after transfer, Western blotting was performed with anti-PPARγ or anti-RXR antibody at a dilution of 1:2,000. Bands were visualized using enhanced chemiluminescence and overnight autorad exposure. IgG bands are displayed as a loading control. **(a) **Single arrowhead denotes high-molecular weight/2 × SUMO1-PPARγ band. **(b) **Double arrowhead denotes high-molecular weight/2 × SUMO1-RXR band; single arrowhead denotes low-molecular weight/1 × SUMO1-RXR band. Lane reference numbers 1P to 8P and 1R to 8R are displayed to facilitate description in the text. IP, immunoprecipitation; LG268, LG100268; Rosi, rosiglitazone; SUMO, small ubiquitin-like modifier; WB, Western blotting.

Maximum inhibition of IL-1β-induced *MMP-1 *and *MMP-13 *expression by LG268 requires 12 to 24 hours of pretreatment with LG268 prior to the addition of IL-1β [21], and we see a similar requirement for pretreatment with rosiglitazone (data not shown). A mechanism of SUMOylation-dependent inhibition of inflammatory gene expression by PPARγ, proposed by Pascual and colleagues [14], suggested a possible explanation for the necessity of pretreatment described above. In their model, SUMOylation of PPARγ anchors the nuclear receptor at the target gene promoter and prevents the clearance of associated corepressors by proinflammatory stimuli [14]. Therefore, we sought to determine whether LG268 and rosiglitazone could cause SUMOylation of RXR and PPARγ in SW-1353 cells. We treated SW-1353 cells with LG268, rosiglitazone, or both compounds for 1 hour, followed by no additional treatment or by IL-1β stimulation for 1 hour. Cell lysates were harvested and total cellular protein was subjected to IP with an antibody against SUMO-1. We then probed the immunoprecipitated fractions for RXR or PPARγ using Western blotting. Figure 9a demonstrates that SW-1353 cells contain a minimal amount of singly SUMOylated PPARγ (molecular weight 82 kDa) and that treatment with IL-1β alone may slightly increase this level (lanes 1P and 2P). However, the presence of rosiglitazone induced a higher molecular weight form of the SUMOylated receptor (lanes 4P and 5P). The molecular weight of the induced band is approximately 115 kDa, which suggests that it is a doubly SUMO1-ylated form of PPARγ. Surprisingly, treatment with LG268 alone (lane 3P) also caused a modest increase in doubly SUMOylated PPARγ, similar to that induced by rosiglitazone (lane 4P). This suggests two conclusions: (a) LG268 treatment alone induces a SUMOylated form of PPARγ, which may transrepress inflammatory genes, and (b) liganding one receptor in the heterodimer can cause SUMOylation of the unliganded partner (for example, liganded RXR causes the SUMOylation of unliganded PPARγ). Interestingly, IL-1β treatment partially decreases the levels of the doubly SUMOylated receptor in all pretreatment groups (lanes 6P, 7P, and 8P), suggesting that proinflammatory stimuli may negatively regulate ligand-induced SUMOylation of PPARγ (see Discussion).

We next investigated whether the rexinoid LG268 similarly affected the SUMOylation state of its receptor target, RXR, which has been shown to be a target of SUMOylation [28]. We performed the same Western blot/IP analysis as described above, with the exception of Western blotting with anti-RXR antibody. As shown in Figure 9b, no SUMOylated RXR is present in untreated cells or in cells treated with IL-1β (lanes 1R and 2R). However, treatment with LG268 led to modest increases in both a low- and high-molecular weight forms of SUMO1-RXR (lane 3R).

The figure also shows that treatment with rosiglitazone induced a high-molecular weight form (molecular weight 115 kDa) of SUMO1-RXR (lane 4R), again likely representing SUMO1-ylation of the receptor at multiple sites. Along with the effect seen in lane 3P of Figure 9b, the SUMOylation of RXR induced by rosiglitazone (lane 4R) lends additional support to the hypothesis that the binding of ligand by one nuclear receptor may cause cross-SUMOylation of the unliganded dimer partner. Treatment with both ligands leads to an increase in the effects observed with single agents: a strong upper band and a modest increase in a lower band (lane 5R). Interestingly, when the cells are stimulated with IL-1β after ligand pretreatment, only the singly SUMO1-ylated form of RXR is seen (lanes 6P, 7P, and 8P). This suggests that treatment with a proinflammatory cytokine may activate pathways that cause removal of SUMO1 from conjugated RXR or perhaps degradation of doubly SUMO1-ylated RXR (see Discussion). However, we can conclude that treatment with either LG268 or rosiglitazone leads to the induction of SUMO1-ylated forms of both PPARγ and RXR.

## Discussion

Previous work has demonstrated that the PPARγ ligand rosiglitazone [17,18,38] and the RXR ligand LG268 [21] each inhibit proinflammatory cytokine induction of *MMP-1 *and *MMP-13 *gene expression. In this study, we address the inhibitory effects of adding both ligands on *MMP-1 *and *MMP-13 *expression in IL-1β-stimulated SW-1353 cells and investigate the mechanisms responsible for this inhibition. We show that rosiglitazone treatment selectively reduces *MMP-1 *and *MMP-13 *mRNA and note that the pattern and magnitude of MMP reduction parallel those seen with LG268 [21]. Additionally, combined treatment results in greater reduction of *MMP-1 *and *MMP-13 *mRNA and hnRNA than those seen with a single ligand. We show that the effect of combined treatment on *MMP-1 *and *MMP-13 *is observed at the protein level, where the addition of both ligands leads to decreased collagen destruction by IL-1β-activated SW-1353 chondrosarcoma cells over single-ligand treatment. Our investigations into the molecular mechanisms of this inhibition centered on the possibility of a shared mechanism since both ligands bind to the RXR:PPARγ heterodimer. We explored the role of RXR:PPARγ binding to the DR-1/AP-1 site in suppressing *MMP-1 *and *MMP-13 *production and addressed possible mechanisms of inhibition, including competitive binding between RXR:PPARγ and AP-1 proteins, altered histone acetylation at the DR-1/AP-1 promoter element, and changes in SUMOylation of PPARγ and RXR.

The competitive binding model identified binding of PPARγ to a degenerate DR-1 site as central to the inhibition of rabbit *MMP-1 *by rosiglitazone, and initial evidence suggested that the inhibition was due to competition between PPARγ and AP-1 for binding at the degenerate DR-1 site [17]. In keeping with this model, we show that, in SW-1353 cells, rosiglitazone and LG268 result in similar levels of consensus DR-1-driven reporter activation, suggesting that treatment with each compound may increase binding to the DR-1 element. However, we did not observe similar results with luciferase reporter constructs driven by the endogenous DR-1/AP-1 elements from the human *MMP-1 *and *MMP-13 *promoters. These data contrast with previous studies in which IL-1β-driven luciferase reporter activity was inhibited in a dose-dependent manner by rosiglitazone; however, those experiments were conducted in rabbit cells transiently transfected with a luciferase construct driven by the rabbit *MMP-1 *promoter [17], whereas the present investigation uses human constructs in human cells. Previously, we noted a discrepancy in the behavior of transiently transfected *MMP-1 *promoter constructs in rabbit [39] versus human [20,21,40] cells in response to treatment with IL-1β. We attribute these differences to a more complex regulation of the human gene and emphasize the importance of measuring expression of the endogenous gene. Since reporter constructs driven by elements of the human *MMP-1 *and *MMP-13 *promoters often do not mirror expression of the endogenous genes, we shifted to a more direct, *in vivo *approach, using ChIP assays to detect changes in PPARγ binding at the DR-1/AP-1 site of the endogenous *MMP-1 *and *MMP-13 *promoters in genomic DNA.

A key aspect of the competitive binding model is mutually exclusive binding of PPARγ and AP-1 transcription factors at the DR-1/AP-1 element, and when considering the competitive binding model, one would expect a decrease in PPARγ binding at the site in cells treated with IL-1β as compared with rosiglitazone- or LG268-treated cells. On the contrary, ChIP analysis using either an HA-PPARγ expression construct (Figure 7) or endogenous PPARγ (Figure 6) detected an increase in PPARγ at the DR-1/AP-1 sites in IL-1β-treated cells, but not rosiglitazone- or LG268-treated cells. These data are inconsistent with the competitive binding model. The large increase in PPARγ at the DR-1/AP-1 site in IL-1β-treated cells was unexpected and may indicate a novel function of PPARγ in IL-1β signaling at the *MMP-1 *and *MMP-13 *promoters. Other studies have implicated IL-1β in regulating expression of PPARγ in chondrocytes, with evidence for both decreasing and increasing PPARγ expression [41,42]. IL-1β inhibits PPARγ mRNA expression in SW-1353 cells, as measured by real-time RT-PCR (data not shown). However, this repression does not affect expression of the PPRE luciferase reporter construct (Figure 5). Therefore, we speculate that PPARγ may be interacting with AP-1 transcription factors at the proximal promoter regions of *MMP-1 *and *MMP-13*. Incorporation of PPARγ in the AP-1 complex may place the nuclear receptor in a position to more efficiently regulate AP-1-driven transcription of *MMP-1 *and *MMP-13*. PPARγ directly interacts with at least one member of the AP-1 transcription factor family [43], and there are examples of other NHRs directly interacting with the AP-1 transcription factors [44-46].

As the PPARγ ChIP did not appear to support the competitive binding model, we next used ChIP to examine the *MMP-1 *and *MMP-13 *promoters for evidence of nuclear receptor-associated coactivator and corepressor activity by detecting changes in histone acetylation. We have previously shown that IL-1β leads to an increase in histone acetylation at these promoters in SW-1353 cells and that this increase in acetylation is blocked when the cells are pretreated with LG268 [21]. Our results show that, as seen previously with LG268, treatment with rosiglitazone prevented histone acetylation at the DR-1/AP-1 site in the proximal promoters of *MMP-1 *and *MMP-13*. Most importantly, dual treatment with rosiglitazone and LG268 resulted in the additive reduction of histone acetylation at both promoters. Given that HDAC activity is typically associated with transcriptional repression [47], this result is consistent with the decrease in *MMP-1 *and *MMP-13 *gene expression in cells treated with a combination of both ligands, suggesting that the compounds may be inhibiting expression of these genes through a common histone acetylation-associated mechanism.

To further investigate this mechanism, we returned to the PPARγ literature. The model describing inhibition of proinflammatory genes by a SUMOylated form of PPARγ proposed by Pascual and colleagues [14] is attractive for several reasons. First, we are investigating the inhibition of MMPs induced by IL-1β, a prototypical inflammatory cytokine. Second, the model requires that the SUMOylated NHR act to anchor the corepressors before they are released by proinflammatory stimuli, possibly providing an explanation for the required pretreatment [21]. Lastly, the mechanism involves regulation via post-translational modification of histones, which suggests that native chromatin conformation is important for the regulation of *MMP-1 *and *MMP-13 *and may help to explain the difficulties seen with transiently transfected luciferase reporter constructs containing endogenous promoter sequences [20,21].

Our data show that treatment with either rosiglitazone or LG268 induces a multiply SUMOylated form of PPARγ (Figure 9a), suggesting that both compounds may work through PPARγ to inhibit proinflammatory genes. This result was rather surprising because, in addition to confirming that a PPARγ ligand can cause SUMOylation of PPARγ in SW-1353 cells, it indicates that treatment with an RXR ligand causes SUMOylation of PPARγ. In addition, we show that LG268 leads to SUMOylation of RXR. This suggests that, similar to rosiglitazone and PPARγ, LG268 inhibits proinflammatory genes by inducing a SUMOylated form of its target receptor, RXR. Interestingly, rosiglitazone also induces SUMOylation of RXR, a result that complements our observation demonstrating LG268-induced SUMOylation of PPARγ. These data may be explained by the fact that, when RXR and PPARγ heterodimerize, they are in close proximity to one another. When the SUMOylation machinery is recruited to the heterodimer through the liganding of one partner, attachment of SUMO may be a somewhat leaky process, thereby leading to SUMOylation of the unliganded dimer partner.

Our data also suggest that, while treatment with LG268 or rosiglitazone induces SUMOylation of RXR, the PPARγ ligand preferentially leads to a higher molecular weight form that is consistent with a doubly SUMOylated version of the receptor. To address this point, we analyzed the protein sequence of RXRα and have identified three putative consensus SUMOylation sites (K201, K245, and K364). The presence of multiple SUMOylation sites on RXR may help to explain the different molecular weight forms of SUMOylated RXR by suggesting that rosiglitazone induces two SUMO molecules to be added to RXR whereas LG268 induces conjugation of only a single SUMO molecule. Because both LG268 and rosiglitazone inhibit IL-1β-induced MMP production and only a singly SUMOylated form of RXR persists when IL-1β is added, perhaps only one site needs to be SUMOylated to cause the inhibitory effect. Interestingly, the K364 site in RXR is very similar in location to the K365 site in PPARγ that Pascual and colleagues [14] demonstrated was required for rosiglitazone-mediated inhibition. Because of the conserved nature of NHR domain structures, K364 may be the required RXR SUMOylation site for inhibition of *MMP-1 *and *MMP-13 *through this mechanism.

## Conclusion

Our data show that the PPARγ ligand rosiglitazone and the RXR ligand LG268 specifically inhibit *MMP-1 *and *MMP-13 *gene expression in IL-1β-stimulated chondrocytic cells. This inhibition appears to be mediated through multiple mechanisms operating at a transcriptional level. Given the link between increased *MMP-1 *and *MMP-13 *gene expression and the arthritides, these compounds could be useful in a therapeutic setting. Clinical investigations using these drugs are already under way in diseases such as diabetes and cancer; rosiglitazone and another thiazolidinedione, pioglitazone, are currently in clinical use as insulin-sensitizing agents in diabetes [48], the rexinoid bexarotene is used as a treatment for cutaneous T-cell lymphoma [49], and there are promising data on the use of LG268 as a chemopreventive compound for breast cancer and lung cancer [50,51]. Combined treatment with ligands for both RXR and PPARγ leads to additive inhibition of *MMP-1 *and *MMP-13 *production, suggesting that these compounds could be used together, in lower doses than single-drug treatment, to reduce or block joint destruction in arthritis, thus minimizing the risk of adverse side effects.

## Abbreviations

AP-1: activator protein-1; ChIP: chromatin immunoprecipitation; DMEM: Dulbecco's modified Eagle's medium; DR-1: direct repeat-1; ECM: extracellular matrix; FBS: fetal bovine serum; FXR: farnesoid X receptor; GAPDH: glyceraldehyde 3-phosphate dehydrogenase; HA: hemagglutinin; HA-PPARγ: hemagglutinin-tagged peroxisome proliferator-activated receptor-gamma; HAT: histone acetyltransferase; HBSS: Hanks' balanced salt solution; HDAC: histone deacetylase; hnRNA: heterogeneous nuclear RNA; IL-1β: interleukin-1-beta; IP: immunoprecipitation; LG268: LG100268; LH: lactalbumin hydrosylate; LXR: liver X receptor; MMP: matrix metalloproteinase; MMPI: matrix metalloproteinase inhibitor; MSS: musculoskeletal syndrome; NHR: nuclear hormone receptor; OA: osteoarthritis; PBS: phosphate-buffered saline; PCR: polymerase chain reaction; PPARγ: peroxisome proliferator-activated receptor-gamma; PPRE: peroxisome proliferator-activated receptor-gamma response element; RA: rheumatoid arthritis; RT: reverse transcription; RXR: retinoid X receptor; SUMO: small ubiquitin-like modifier; TCA: trichloracetic acid.

## Competing interests

The authors declare that they have no competing interests.

## Authors' contributions

PSB and ACS were responsible for study design, acquisition of data, analysis and interpretation of data, manuscript preparation, and statistical analysis and contributed equally to this work. YR was responsible for acquisition of data, analysis and interpretation of data, and manuscript preparation. MBS was responsible for study design and manuscript preparation. CEB was responsible for study design, analysis and interpretation of data, and manuscript preparation. All authors read and approved the final manuscript.
